# Changes in Oxidative Stress and Intestinal Permeability during Pregnancy in Women with Gestational Diabetes Mellitus Treated with Metformin or Insulin and Healthy Controls: A Randomized Controlled Trial

**DOI:** 10.3390/antiox12111981

**Published:** 2023-11-08

**Authors:** Andrea Fernández-Valero, Nerea Peña-Montero, Fuensanta Lima-Rubio, Carolina Gutiérrez-Repiso, Teresa María Linares-Pineda, María José Picón-César, Raquel Sancho-Marín, Francisco J. Tinahones, Sonsoles Morcillo, María Molina-Vega

**Affiliations:** 1 Department of Endocrinology and Nutrition, Hospital Universitario Virgen de la Victoria de Málaga, 29010 Málaga, Spainmolinavegamaria@gmail.com (M.M.-V.); 2 Department of Medicine and Dermatology, Málaga University, 29010 Málaga, Spain; 3 Laboratory of the Biomedical Research Institute of Málaga, Virgen de la Victoria University Hospital, Universidad de Málaga, 29010 Málaga, Spain; 4 Centro de Investigación Biomédica en Red (CIBER) de Fisiopatología de la Obesidad y Nutrición, Instituto Salud Carlos III, 28029 Madrid, Spain

**Keywords:** gestational diabetes mellitus, oxidative stress, intestinal permeability, metformin

## Abstract

Both oxidative stress and intestinal permeability are increased in hyperglycemic situations and have been shown to be reduced by metformin in type 2 diabetes mellitus (T2DM) patients. The aim of this study was to elucidate the effect of metformin on oxidative stress and intestinal permeability in women with gestational diabetes mellitus (GDM) treated with metformin compared to those treated with insulin and healthy controls. A total of 120 women were included from August 2016 to February 2022: 41 received metformin (MET group), 38 received insulin (INS group), and 41 were healthy controls. Baseline and antenatal visits were carried out at 25.4 ± 4.8 and 36.1 ± 0.8 weeks of pregnancy, respectively. Advanced oxidation protein products (AOPPs), total antioxidant capacity (TAC), and zonulin levels were measured at every visit. Zonulin levels from baseline to prepartum visit increased significantly in both healthy controls (0.6 ± 0.9 to 1.2 ± 1.7 ng/mL, *p* = 0.004) and the INS group (0.4 ± 0.3 to 0.6 ± 0.5 ng/mL, *p* = 0.034) but did not significantly change in the MET group (0.4 ± 0.4 to 0.5 ± 0.4 ng/mL, *p* = 0.202). However, TAC and AOPP levels significantly increased in women with GDM, both in the INS and MET groups but not in the healthy controls. In conclusion, in our population, metformin has been shown to avoid an increase in intestinal permeability but failed to avoid an increase in oxidative stress related to hyperglycemia.

## 1. Introduction

Gestational diabetes mellitus (GDM) is defined as any condition of impaired glucose tolerance with onset during pregnancy [1]. Is the most prevalent metabolic complication of pregnancy and it is associated with a well-documented range of adverse outcomes for both mother and newborn during pregnancy and after delivery [2]. Lifestyle modifications, including dietary changes and abandoning a sedentary lifestyle, are the first lines of treatment. Despite this, up to 30% of pregnant women will not achieve adequate glucose control and will require the addition of pharmacological therapy [3]. Although traditionally insulin has been the only pharmacological treatment for GDM, in the last few years, metformin has been increasingly used. It has been included in GDM guidelines as an option of treatment [4] as a safe pharmacological alternative to insulin [3], or even as the first-line treatment in some cases [5]. There is strong evidence supporting metformin as a safe therapy with some advantages over insulin in the treatment of gestational diabetes [6,7,8]. The MeDiGes study was an open label randomized clinical trial enrolling 200 pregnant women with GDM who needed pharmacological treatment performed by our group. We evaluated metformin against insulin and found that the metformin-treated group had lower gestational weight gain, lower postprandial glycemia, and diminished hypoglycemia events compared to the insulin group. Regarding obstetrical outcomes, fewer cesarean deliveries were recorded in the metformin group [9]. Even though up to 46.3% of women will need to add supplementary insulin to achieve normoglycemia, the beneficial effect on reducing maternal weight gain is maintained, showing metformin to be worth using even when used in combination [10].

Hyperglycemia induces reactive oxygen species (ROS) production, which can cause tissue damage and cellular alterations [11]. Exogenous and endogenous antioxidants neutralize ROS to counteract their harmful effects and maintain homeostasis. Exacerbated ROS production under hyperglycemic conditions can overwhelm the antioxidant response and result in oxidative stress (OS), which has been implicated in the pathogenesis and progression of type 2 diabetes mellitus (T2DM) [11,12]. During pregnancy, there is a process of low-grade oxidative stress considered physiological. However, despite the significant role of ROS in this period, the high glucose concentrations in GDM result, such as in T2DM, in excessive ROS production contributing to oxidative stress, cell, and tissue damage [13]. Moreover, ROS hyperproduction causes a reduction in insulin secretion through damage to pancreatic islet cells and can also impair insulin signaling, resulting in insulin resistance (IR). Both the impairment of β-cell function and IR lead to persistent hyperglycemia [11]. Studies of T2DM have been critical in elucidating the role of oxidative stress in diabetes. However, data available in relation to GDM and their correlation with maternal and neonatal outcomes is limited [14,15].

The intestinal microbiota undergoes several changes during pregnancy, causing dysbiosis with a profile similar to people with metabolic syndrome [13]. Due to dysbiosis, there is an increase in intestinal permeability, regulated by junction proteins, such as zonulin, in which serum levels are increased in GDM [16]. This increase in permeability can favor the movement of LPS (which are strong inducers of inflammation) from the intestine to the circulation, negatively regulating insulin signaling and promoting even more insulin resistance [17]. This makes microbiota a crucial therapeutic target for GDM, both preventively and for treatment [13]. Multiple reports provide evidence that metformin can modify the composition of gut microbiota [18] and that markers of oxidative stress significantly decrease in patients with T2DM treated with metformin [19], but if the same effects are found in pregnant women with GDM, they must be elucidated.

Therefore, the aim of this study was to evaluate the effect of treatment with metformin in women with GDM on the oxidative stress and intestinal permeability parameters compared with insulin and with healthy pregnant women, with the purpose of enhancing comprehension of the action of metformin in the unexplored context of GDM.

## 2. Materials and Methods

### 2.1. Study Population

In this study (funded by Consejería de Salud y Familias, Junta de Andalucía, PI-0419-2019), 120 pregnant women who attended the Diabetes and Pregnancy Unit of Virgen de la Victoria University Hospital (Málaga, Spain) between August 2016 and February 2022 after a positive screening with a 50 g 1 h glucose load test were included. From August 2016 to January 2019, in the context of the MeDiGes Trial (European Union Clinical Trials Registry: EudraCT 2015-000361-31), 82 women diagnosed with GDM who were unable to achieve adequate glucose control with lifestyle changes and required the addition of pharmacological treatment were randomized to be treated with insulin or metformin [9]. In addition, from January 2019 to February 2022, 41 pregnant women not diagnosed with GDM were included to participate as healthy controls.

Inclusion criteria were the following: age between 18 and 45, being in the second or third trimester of pregnancy, singleton pregnancy, and being able to give informed consent. On the other hand, the exclusion criteria were being in the first trimester of pregnancy, multiple gestation, a fasting glycemia > 120 mg/dL (6.6 mmol/L), or the presence of a language barrier.

### 2.2. Study Protocol

A two-step strategy according to the National Diabetes Data Group (NDDG) criteria was used to diagnose GDM. First, between the 24th and 28th weeks of gestation, a screening with a 50 g oral glucose load (O’Sullivan test) followed by a blood sampling one hour later was performed. Pregnant women with a post-load glucose value equal to or greater than 140 mg/dL (7.8 mmol/L) underwent a diagnostic 100 g, 3 h oral glucose tolerance test (OGTT). The thresholds for the diagnosis were 105 mg/dL (5.8 mmol/L) for fasting glucose, 190 mg/dL (10.6 mmol/L) at 60 min, 165 mg/dL (9.2 mmol/L) at 120 min, and 145 mg/dL (8.0 mmol/L) at 180 min. If two or more values were equal or greater than these levels, a diagnosis of GDM was performed [20]. After being diagnosed with GDM, women were recommended to make lifestyle modifications (including dietary changes and exercise) and self-blood glucose monitoring (SBGM) four times a day (at fasting and one hour postprandial). After one week, the endocrinologist analyzed the glycemic controls. When two or more fasting glucose values were ≥95 mg/dL (5.3 mmol/L) and/or two or more one-hour postprandial glucose values were ≥140 mg/dL (7.8 mmol/L), it was considered a failure of lifestyle changes alone. At that time the addition of pharmacological treatment was recommended, and women were requested to be included in the study. Those who agreed to enroll in the study after signing the informed consent were randomized to receive metformin (MET group) or insulin (INS group). If adequate glycemic control was not achieved with metformin, insulin was added to the treatment.

On the other hand, pregnant women whose OGTT was normal and GDM was ruled out were requested to be included as healthy controls.

For this study, basal and prepartum visits (at 36–37 weeks of gestation) were carried out. Women completed a structured interview at the basal visit that included the following data: age, family history of diabetes, obstetrical and gynecological history, presence of previous or current medical conditions, and current treatment. On both visits, antecubital venous blood samples were collected, and weight and blood pressure were measured. Height measurements were taken only at the basal visit.

The CONSORT checklist has been filled out in Table S1.

### 2.3. Oxidative Stress and Intestinal Permeability Analysis

At basal and prepartum visits, maternal antecubital venous blood samples were collected. The serum was separated and immediately frozen at −80 °C until analysis.

Serum levels of zonulin were determined by ELISA (Human zonulin ELISA kit, Cusabio^®^ Technology Llc, Houston, TX, USA). Total antioxidant capacity (TAC) was determined by an OxiSelect™ Total Antioxidant Capacity (TAC) Assay Kit (Cell Biolabs, San Diego, CA, USA), and the advanced oxidation protein product (AOPP) was measured by an OxiSelect™ AOPP Assay Kit (Cell Biolabs, INC).

### 2.4. Statistical Analysis

Data were analyzed using IBM SPSS (15.0 version for Windows; SPSS, Chicago, IL, USA), and statistical significance was set at *p* < 0.05. An ANOVA test was used to compare the quantitative data and a chi-squared test was used to compare the qualitative data. Data are shown as mean ± standard deviation or percentage.

### 2.5. Ethics

This study was conducted in accordance with the principles of the Declaration of Helsinki. It was reviewed and approved by the Ethics and Research Committee of the Virgen de la Victoria University Hospital (Málaga, Spain). After being fully informed of the characteristics of the study, all the participants signed a written informed consent.

## 3. Results

### 3.1. Study Population

**Figure 1:**
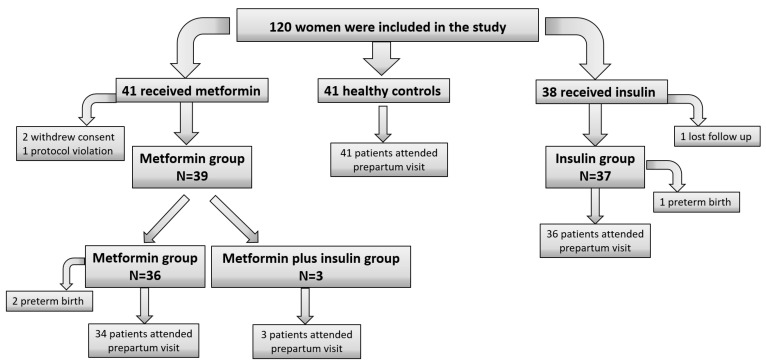
Flow diagram.

A total of 120 pregnant women were included in this study. There were 41 healthy controls (control group), and all of them attended the prepartum visit. There were 79 women with GDM who did not achieve adequate glycemic control with lifestyle modifications and were required to add pharmacological treatment, of whom 41 were randomized to the MET group and 38 were randomized to the INS group. In the MET group, after one withdrawal of consent and one protocol violation, 39 subjects continued the study. In the INS group, after one was lost to follow-up, thirty-seven subjects continued the study. Only three subjects in the MET group required the addition of insulin to reach glycemic goals during the follow-up. Due to two preterm births in the MET group and one preterm birth in the INS group, thirty-seven subjects in the MET group and thirty-six subjects in the INS group attended the prepartum visit (Figure 1).

### 3.2. Characteristics of the Study Population

In Table 1, we show the characteristics of the study population and comparisons between groups at baseline, at prepartum visit and regarding obstetric and perinatal outcomes. The mean baseline gestational age was 25.4 ± 4.8 weeks and the prepartum visit mean gestational age was 36.1 ± 0.8 weeks.

At baseline, we found a lower BMI in healthy controls compared with the other two groups. As expected, women with GDM had a more frequent previous history of GDM and macrosomic newborns compared with the control group. Regarding weight change, no difference was found in total weight gain during pregnancy among groups. However, if we focus on the weight gain from enrollment in the study to the prepartum visit, it was significantly lower in the MET group.

Regarding lipid profile, we observed healthy controls to have higher total cholesterol (TC) and LDL cholesterol (LDL-c) in both baseline and prepartum levels and higher HDL cholesterol (HDL-c) at baseline in comparison with the GDM group. No significant differences were found in triglycerides (TGs).

In terms of glycemic control, we found that mean postprandial glycemia was significantly lower in the MET group compared with the INS group (121.6 ± 8.1 mg/dL vs. 130.3 ± 12.6 mg/dL; *p* = 0.001). Non-significant differences in the prepartum HbA1c or fasting glycemia were found. At the prepartum visit, the mean dose of metformin was 1493.5 ± 550.2 mg, and the mean total daily dose of insulin was 36.7 ± 29.5 IU. When comparing data from perinatal outcomes, there was a significantly higher birthweight percentile in the INS group compared to the healthy control group.

### 3.3. Oxidative Stress and Intestinal Permeability Parameters

In Table 2, we show the oxidative stress parameters and zonulin in each group at baseline and prepartum level. We also assessed the changes in each group during pregnancy (from baseline to prepartum level) and compared the results to see the differences between groups.

No significant differences were observed in baseline levels of TAC, zonulin, or AOPPs between groups. In the prepartum visit, healthy controls had higher levels of zonulin compared with pregnant women with GDM with metformin and insulin treatment. The change in zonulin levels from basal to prepartum was higher in the controls compared with the MET group. Nevertheless, it was similar between both GDM groups and between the INS group and controls.

The changes in the studied parameters during the follow-up are shown in Table 3. In the control group, there was a significant increase in zonulin levels; however, no significant changes in AOPPs or TAC were observed. In the INS group, there was a significant increase in all parameters studied. In the MET group, there was a significant increase in AOPPs and TAC, although no significant changes in zonulin were observed.

## 4. Discussion

In this randomized clinical trial of pregnant women with GDM treated with metformin or insulin compared to a control group of healthy pregnant women, we found that oxidative stress parameters (AOPP and TAC) increased in both GDM women treated with insulin and metformin but not in healthy controls. However, while intestinal permeability (zonulin) increased during the follow-up in both healthy controls and women with GDM treated with insulin, it remained the same in women with GDM treated with metformin.

During pregnancy, the increase in metabolic rate necessary to ensure adequate fetal growth and development comes with increased OS in the placenta [21]. At moderate or low levels, ROS stimulates cellular responses and immune function, thus playing a key role in maintaining normal cellular homeostasis [11]. Despite its important physiological role, the overproduction of ROS can cause structural modifications in proteins, lipids, and nucleic acids and consequently alter their function [22]. In pregnancy, increased OS is counterbalanced by an increase in the antioxidant defense system with increased antioxidant enzymes in the placenta. However, when OS exceeds the antioxidant defense in the placental tissues, oxidative damage can spread to distal tissues [21]. There is growing evidence that OS can play a key role in the pathogenesis of GDM since studies have found enhanced oxidation products and reduced antioxidant capacity in these patients [23,24,25]. Hyperglycemia in pregnant women causes an increase in ROS production via the auto-oxidation of glucose and non-enzymatic protein glycation [26].

AOPPs are a group of oxidized proteins that can be used as a biomarker of OS since proteins are the main target of oxidants [27]. AOPPs have been found to have increased in patients with T2DM and its complications in several studies. Few studies have assessed AOPPs in GDM. Li et al. [24] observed elevated levels of AOPPs in patients with GDM at various gestational ages compared to normal subjects, suggesting that oxidative damage may be increased. Moreover, they found that levels were increased even before GDM diagnosis (at 16–20 weeks of gestation), suggesting that increased oxidative stress may contribute to the development and progression of GDM. Karacay et al. [28] found that circulating AOPPs increased at 24–36 weeks of pregnancy in GDM compared to non-complicated pregnancies. Similar results were found by Yarsilikal et al. [27]. The AOPP was significantly increased in women with GDM compared to those in whom gestational diabetes had been ruled out by a normal glucose tolerance test. However, in our study, we did not find significant differences in the levels of the AOPP between GDM and healthy controls, but we observed a significant increase in the AOPP from baseline to prepartum in both groups of GDM patients, which was not present in healthy controls.

Regarding the antioxidant system, antioxidants are known to protect against the harmful effects of ROS. Among the antioxidant agents, we can find superoxide dismutase (SOD), catalase (CAT), vitamin C, vitamin E, beta-carotene, and glutathione peroxidase (GPX) [29]. Due to the cumulative activity of all of them, instead of evaluating each agent separately, it is worth measuring the unified activity of all the agents with the total antioxidant capacity (TAC) level. This biomarker allows us to measure the antioxidant potential of all fluids of an organism [30].

In the present study, we observed that TAC increased significantly from baseline to the prepartum levels in GDM, but not in healthy controls. Not many studies have evaluated the changes in the levels of these markers over the course of pregnancy. De Lucca et al. [23] found an increase in the levels of TAC in non-complicated gestation in the third trimester compared with the first trimester, but not compared with the second trimester. As we only assessed the last period of pregnancy, we think it might be the reason why we do not see that increase in our healthy population. However, it can be observed that although not significant, both baseline and prepartum levels of TAC were lower in the GDM group than in the control group, as previously described by other studies. In the Parast study [29], they observed that serum levels of TAC in pregnant women at 24–28 weeks of gestation with GDM were significantly lower as compared to a healthy group (2.3 ± 0.7 μmol/L vs. 3.7 ± 0.1 μmol/L; *p* < 0.001). Shang et al. [25] studied 68 women and detected a decrease in TAC (*p* < 0.05) in the plasma and placenta samples of those diagnosed with GDM compared to their respective healthy controls, while Karacay et al. [28] found similar results with total antioxidant status (TAS). Kapustin et al. [30] evaluated pregnant women with different types of DM (including T1DM, T2DM, and GDM) at the first and third trimesters considering the treatment and preconception planning, and they found that TAC levels manifested a noteworthy decrease in all groups compared to the controls, suggesting antioxidant system decompensation in long-term hyperglycemia. However, there are discrepancies concerning the expression and activity of antioxidants in GDM, and contrary data were obtained in other studies, mainly when salivary TAC was evaluated.

In a study of 68 pregnant women, Zygula et al. [31] showed decreased TAC levels in plasma and saliva in those with GDM. Similar results were reported by Zamani-Ahari et al. [32] and Surdacka et al. [33] showing higher salivary TAC levels in the GDM group compared to non-diabetic pregnant women. They proposed that this might be because the increase in OS observed in GDM was counteracted.

The role of metformin in the alleviation of OS in T2DM patients has been shown in previous reports. It exerts this beneficial role through several mechanisms: the trapping of hydroxical radicals, enhancing the endogenous antioxidant system (including glutathione reductase, CAT and SOD, or GSH content), and downregulating NADPH oxidase (one of the major producers of cellular ROS) [19,34]. In a study of 40 newly diagnosed medication-naive T2DM patients, Adeshara et al. [26] found that the AOPP and LPO were reduced after metformin therapy compared to baseline levels (*p* < 0.001). On the other hand, antioxidants (GSH, CAT, and PON-1) were noticeably increased. Despite this, in our study, we did not find this expected effect in reducing oxidative markers since in both insulin- and metformin-treated GDM patients, we found an increase in the AOPP. Regarding antioxidants, there was an increase in TAC from baseline to prepartum in both GDM groups, but even though not significant, the change in TAC was higher in the metformin group, in accordance with previous studies.

Concerning intestinal permeability, it is known that during pregnancy, the intestinal microbiota undergoes several changes, causing dysbiosis [13]. Dysbiosis produces an increase in intestinal permeability regulated by zonulin, among others. The stimulation of a zonulin release leads to the dissociation of the zonula occludens-1 protein from tight junctions between intestinal epithelial cells, resulting in increased intestinal permeability [35], which can favor the movement of LPS, which are strong inducers of inflammation [17]. Mokkala et al. [36] reported an increase in serum zonulin concentration from early to late pregnancy, suggesting that intestinal permeability is increased as pregnancy proceeds. Previous studies, such as Daneshvar et al. [16] or Guvey et al. [35], have demonstrated the relationship between zonulin and GDM, showing higher serum zonulin levels in women with GDM compared to non-diabetic pregnant women. Our data showed a significant increase in zonulin during the follow-up in both healthy controls and GDM women treated with insulin; nevertheless, it remained the same in those women with GDM treated with metformin, probably due to the beneficial changes in the composition of gut microbiota. Several studies have revealed that metformin affects the composition of gut microbiota; in fact, previously, our group analyzed the changes in gut microbiota in metformin-treated GDM patients [37], showing a decrease in phylogenetic diversity, an increase in *Proteobacteria*, *Enterobacteriaceae,* and *Coprococcus catus,* and a reduction in the abundance of *Firmicutes* and *Peptoestreptococcaceae,* which could mediate its clinical benefits. It has been described an increase in the abundance of *Akkermansia muciniphila* promoting mucin production and thereby increasing the mucus layer, which acts as a barrier for LPS [38]. Furthermore, studies have reported an increase in the expression of MUC2 after metformin treatment, which leads to the recovery of tight-junction proteins, such as zonulin-1 and occludin, and, therefore, a reduction in intestinal permeability, suggesting that the gut is a major target of metformin action [18].

Regarding lipids, a physiological rise is produced during pregnancy in order to favor the correct growth and development of the fetus, with the most severe alterations in the second and third trimesters [39]. Although it has been previously described that women with GDM had higher levels of TGs, TC, and LDL-c and lower levels of HDL-c, compared to non-GDM women [40], our results are different. In our population, we have found healthy controls to have higher levels of TC and LDL-c in both baseline and prepartum visits and higher HDL-c at baseline compared with women with GDM independent of treatment, with no statistically significant differences in TGs. A possible explanation for this is that maybe women finally diagnosed with GDM due to their higher BMI since the beginning of pregnancy could have been given special advice to do a healthier diet and physical activity.

Our study has some important strengths. To the best of our knowledge, this is the first study analyzing the effect of treatment with metformin in women with GDM on reducing oxidative stress and intestinal permeability compared with insulin and healthy controls. Despite not having a large sample size, our study is similar to or even larger than other similar studies, and the study population was well-characterized and had good compliance. As a limitation, we only studied the last period of pregnancy after an OGTT was performed; it would be interesting to assess and compare the changes from the first trimester to the prepartum.

## 5. Conclusions

In conclusion, in this randomized clinical trial of pregnant women with GDM treated with metformin or insulin compared to a group of healthy pregnant women, metformin showed a beneficial effect over intestinal permeability. It is probably mediated by its positive effect on gut microbiota, but it failed to show a consistent benefit in oxidative stress parameters in our population. Further studies are required in order to deepen the physiologic mechanisms implicated in the benefits observed with metformin in the treatment of GDM.

## Figures and Tables

**Table 1:** Clinical characteristics of the study population at baseline, at prepartum visit and regarding obstetric and perinatal outcomes.

	Healthy Controls	INS Group	MET Group	*p*-Value
**Baseline**	**N = 41**	**N = 38**	**N = 41**	
Gestational age	27.1 ± 1.6	23.1 ± 7.1	23.4 ± 6.0	
Age (years)	33.7 ± 4.2	34.5 ± 4.9	34.9 ± 4.9	0.477
Pre-pregnancy BMI (kg/m^2^)	26.1 ± 5.2 ^a^	30.7 ± 4.9 ^b^	28.9 ± 5.4 ^b^	**0.001**
Nulliparous (%)	55.8	18.6	25.6	**0.001**
GDM in a previous pregnancy (%)	5.6	44.4	50	**0.005**
Previous macrosomia (%)	16.7	44.4	38.9	**<0.001**
Family history of DM (%)	23.6	40	36.4	0.055
Fasting glycemia (mg/dL)	75.6 ± 7.1 ^a^	85.5 ± 11.4 ^b^	84.1 ± 12.7 ^b^	**<0.001**
HbA1c (%)	5.1 ± 0.3 ^a^	5.4 ± 0.4 ^b^	5.3 ± 0.4 ^b^	**0.007**
Total cholesterol (mg/dL)	264.6 ± 42.2 ^a^	222.6 ± 45.4 ^b^	220.8 ± 54.5 ^b^	**<0.001**
HDL cholesterol (mg/dL)	81.5 ± 17.9 ^a^	70.9 ± 14.7 ^b^	67.0 ± 14.5 ^b^	**<0.001**
LDL cholesterol (mg/dL)	145.7 ± 36.4 ^a^	113.5 ± 38.0 ^b^	118.1 ± 37.4 ^b^	**<0.001**
Triglycerides (mg/dL)	192.4 ± 79.2	194.1 ± 63.8	200.3 ± 78.8	0.457
Ferritin (ng/mL)	18.0 ± 19.2	26.2 ± 24.2	19.1 ± 17.0	0.183
**Prepartum Visit**	**N = 41**	**N = 36**	**N = 37**	
Gestational weight gain (kg)	11.0 ± 5.1	9.3 ± 5.5	8.0 ± 5.9	0.063
Weight gain from enrollment to prepartum visit (kg)	4.6 ± 3.0 ^a^	4.2 ± 4.1 ^a^	1.7 ± 3.8 ^b^	**0.002**
Mean fasting glycemia during follow-up		92.4 ± 7.8	90.3 ± 6.6	0.214
Mean postprandial glycemia during follow-up		130.3 ± 12.6	121.6 ± 8.1	**0.001**
Mean prepartum fasting glycemia (mg/dL)	73.7 ± 10.1	77.7 ± 11.4	76.3 ± 10.2	0.250
HbA1c (%)	5.3 ± 0.3	5.5 ± 0.4	5.4 ± 0.4	0.316
Total cholesterol (mg/dL)	267.1 ± 41.9 ^a^	238.4 ± 44.2 ^b^	239.7 ± 45.3 ^b^	**0.006**
HDL cholesterol (mg/dL)	78.5 ± 21.7	72.8 ± 15.4	70.6 ± 14.4	0.134
LDL cholesterol (mg/dL)	141.7 ± 35.4 ^a^	118.9 ± 34.4 ^b^	116.3 ± 34.3 ^b^	**0.003**
Triglycerides (mg/dL)	248.2 ± 85.3	236.1 ± 72.1	268.4 ± 91.8	0.257
Ferritin (ng/mL)	18.8 ± 21.0	12.9 ± 8.7	12.8 ± 7.2	0.129
**Obstetric and Perinatal Outcomes**				
Induction of labor (%)	30.8	38.5	30.8	0.331
Type of delivery (%)				0.088
- Non-instrumental vaginal	47.3	36.1	68.4	
- Instrumental	8	8.4	5.3	
- Cesarean	44.7	55.5	26.3	
Birthweight (gr)	3238.9 ± 418.6	3379.9 ± 495.2	3300.9 ± 575.8	0.502
Birthweight percentile	46.7 ± 24.9 ^a^	65.8 ± 32.3 ^b^	58.0 ± 32.1 ^a,b^	**0.036**
Birth length (cm)	49.9 ± 2.1	49.9 ± 2.8	50.3 ± 1.8	0.635
Head circumference (cm)	34.5 ± 3.1	34.0 ± 1.9	34.5 ± 1.9	0.539

Data are shown as mean ± standard deviation or percentage. BMI: body mass index; GDM: gestational diabetes mellitus: DM: diabetes mellitus; HDL: high-density lipoprotein; LDL: low-density lipoprotein. Different superscripted letters mean significant differences (*p* < 0.05) according to an ANOVA followed by Duncan’s post hoc test.

**Table 2:** Comparison between groups regarding oxidative stress parameters and zonulin.

	Healthy Controls	INS Group	MET Group	*p*-Value
Baseline TAC (µM CRE)	1710.0 ± 197.9	1630.7 ± 268.4	1657.4 ± 182.6	0.294
Baseline zonulin (ng/mL)	0.6 ± 0.9	0.4 ± 0.3	0.4 ± 0.4	0.437
Baseline AOPP (µM)	211.0 ± 151.8	146.2 ± 52.0	157.4 ± 83.2	0.050
Prepartum TAC (µM CRE)	1814.9 ± 227.5	1707.3 ± 281.6	1778.9 ± 256.9	0.200
Prepartum zonulin (ng/mL)	1.2 ± 1.7 ^a^	0.6 ± 0.5 ^b^	0.5 ± 0.4 ^b^	**0.018**
Prepartum AOPP (µM)	225.7 ± 130.8	180.5 ± 88.2	220.9 ± 113.5	0.166
TAC change (µM CRE)	104.9 ± 172.5	76.6 ± 205.1	121.5 ± 187.9	0.608
Zonulin change (ng/mL)	0.6 ± 1.1 ^a^	0.2 ± 0.5 ^a,b^	0.1 ± 0.5 ^b^	**0.038**
AOPP change (µM)	45.4 ± 70.3	34.2 ± 78.1	63.5 ± 89.8	0.306

TAC: total antioxidant capacity; AOPPs: advanced oxidation protein products; CRE: copper reducing equivalent. Different superscripted letters mean significant differences (*p* < 0.05) according to an ANOVA followed by Duncan’s post hoc test.

**Table 3:** Changes in oxidative stress parameters and zonulin during the follow-up.

	Baseline	Prepartum	*p*-Value
**Total**			
TAC (µM CRE)	1688.0 ± 218.9	1767.0 ± 254.9	**0.001**
Zonulin (ng/mL)	0.5 ± 0.6	0.8 ± 1.1	**<0.001**
AOPP (µM)	173.6 ± 110.3	210.1 ± 114.0	**0.001**
**Healthy Controls**			
TAC (µM CRE)	1710.0 ± 197.9	1814.9 ± 227.5	0.389
Zonulin (ng/mL)	0.6 ± 0.9	1.2 ± 1.7	**0.004**
AOPP (µM)	211.0 ± 151.8	225.7 ± 130.8	0.551
**Ins Group**			
TAC (µM CRE)	1630.7 ± 268.4	1707.3 ± 281.6	**0.037**
Zonulin (ng/mL)	0.4 ± 0.3	0.6 ± 0.5	**0.034**
AOPP (µM)	146.2 ± 52.0	180.5 ± 88.2	**0.015**
**Met Group**			
TAC (µM CRE)	1657.4 ± 182.6	1778.9 ± 256.9	**0.001**
Zonulin (ng/mL)	0.4 ± 0.4	0.5 ± 0.4	0.202
AOPP (µM)	157.4 ± 83.2	220.9 ± 113.5	**<0.001**

TAC: total antioxidant capacity expressed in µM CRE; AOPPs: advanced oxidation protein products expressed in µM; Zonulin expressed in ng/mL.

## Data Availability

Data are available upon request from the authors.

## Supplementary Materials

The following are available online at https://www.mdpi.com/article/10.3390/antiox12111981/s1: Table S1: CONSORT 2010 checklist of information to include when reporting a randomized trial.
